# Supplementary material for: Understanding older adults’ perception, acceptance, and adoption of smart home technologies
Source: PLoS One. 2026 Apr 21;21(4):e0345563. doi: 10.1371/journal.pone.0345563 (PMC13098919; doi:10.1371/journal.pone.0345563)
Supplement: S1 Appendix — (PDF) [file pone.0345563.s001.pdf]

Supporting information

S1 Appendix. Interview format and collated data

S1 Table. Interview Topics and Questions.

Topic	Questions
QoL	1. Meaning of Quality of Life.<br>2. Participants QoL status.<br>3. COVID-19 Impact on QoL.<br>4. Knowledge and discussion about Fig 2 in [1].
General Technology and Clinical Devices Related Questions	1. Do you use any clinical, assistive or medical devices to monitor your health? (if answered yes then further questions were asked which are listed below in this category)<br>2. What kind of clinical, assistive or medical devices do you use to monitor your health?<br>3. Do you have any difficulty using these devices?<br>4. Do you require any help in operating or installing these devices?<br>5. How frequently do you use these devices?<br>6. Do you share the obtained data with anyone else such as a family member or your GP or a doctor?<br>7. Did someone encourage you to buy these devices?<br>8. Do these devices fulfil their intended purpose?

Topic	Questions
Smart Technology and Solutions in Tables **??** and **??**	1. What is the level of awareness and knowledge among participants?<br>2. What is the current adoption rate of smart technologies among participants?<br>3. What are the technical, ethical or other challenges regarding smart technologies faced by participants?<br>4. What are the opinions, perceptions and concerns among participants?<br>5. What is the willingness to adopt these technologies?<br>6. Does awareness impact their willingness to adopt these technologies?<br>7. How does trust in technology translate to adoption?
Personal	1. Gender<br>2. Age<br>3. Country<br>4. Health status or condition<br>5. Living status (with partner/alone/more than 3 members in the family)<br>6. What is your physical activity level? (Sedentary, Moderate, High)<br>7. What kind of activities or exercises do you do to keep yourself active?
Health and Safety	1. Have you faced any emergency situations where you needed help (for example a fall)?<br>2. What kind of emergency situations have you faced before?<br>3. Did you get a prompt response in the given case of an emergency? (experience with response)<br>4. Do you worry about your health and safety when you are alone?<br>5. Do you face any challenges performing any activities of daily life such as climbing the stairs, transitioning from sitting to standing position, doing chores etc?

Topic	Questions
Socialising	1. How often do you socialise? (every day, once a week, once every 2 weeks, once a month+)<br>2. Do you use any communication technology for socialising? (e.g. Facebook, Skype)?
Other	1. Would you prefer having a camera monitoring system that does not store images and videos but is only used to monitor your health?<br>2. Would you prefer to have control over sharing your data with GP or family or keep it just to yourself?<br>3. Has COVID-19 changed your perception about any of the technologies discussed during this interview?
Survey Questionnaire (at the end of the interview using Qualtrics)	1. What is the reason for hesitation in adopting technology from your perspective? (Results shown in S3 Table and Fig 8)<br>2. What is your affordability range for the various smart technologies discussed in this interview? (Results shown in Fig 6 and 7)<br>3. Other questions related to adopting technology and its results are presented in S2 Table, Fig 3 and 4.

S2 Table. Smart home technology related questions, with SH: Smart Home, SP: Smart Phone, SW:Smart Clothes/Wearables, C:Cameras, O: Other.

Question	SH	SP	SW	C	O
Which technology do you own currently?	12%	45%	2%	12%	29%
Which one are you likely to adopt in future?	48%	16%	12%	20%	4%
Which technology makes you think has privacy concerns?	14%	23%	14%	46%	5%
Which technology do you think provides fewer options to control technology?	33%	0%	22%	44%	0%
Which technology do you think has the most beneficence? (the benefit and welfare of the person or provides the intended benefits to the person who is using the technology.)	35%	46%	4%	4%	12%
Which technology do you think is unobtrusive? (doesn't become an obstacle or a block in the path)	39%	44%	6%	6%	6%
Which technology do you think promotes independence?	38%	38%	14%	5%	5%
Which technology do you think has autonomy? (Ability to act freely in accordance with a self-chosen plan)	42%	37%	5%	5%	11%
Which technology do you think supports Socialising? / support social relations and the capacity to interact with the external social, digital and natural environments	16%	72%	0%	4%	8%

S3 Table. Reasons for Hesitation in Adopting Technology.

Reasons	1	2	3	4	5	6	7	8
Privacy	27%	23%	23%	14%	5%	9%	0%	0%
Control over technology	14%	27%	23%	32%	0%	0	0%	5%
Beneficence (provides the intended benefits of the technology.)	14%	18%	23%	32	5%	9%	0%	0%
Unobtrusive (does not become an obstacle)	0%	5%	9%	14%	55%	5%	14%	0
Autonomy (promotes independence)	14%	9%	14%	0%	14%	46%	5%	0
Supports Socialising	9%	5%	9%	5%	5%	27%	36%	5%
Affordability (cost)	23%	14%	0%	5%	9%	5%	36%	9
Discomfort	0%	0%	0%	0%	9%	0%	9%	82%

The table lists reasons for hesitation in adopting technology, with each reason ranked from 1st to 8th order based on participants' preference.
Note: $1 \rightarrow 1^{st}$Order, $2 \rightarrow 2^{nd}$Order, $3 \rightarrow 3^{rd}$Order, $4 \rightarrow 4^{th}$Order, $5 \rightarrow 5^{th}$Order, $6 \rightarrow 6^{th}$Order, $7 \rightarrow 7^{th}$Order, $8 \rightarrow 8^{th}$Order

References

1. Pirzada P, Wilde A, Doherty GH, Harris-Birtill D. Ethics and acceptance of smart homes for older adults. Informatics for Health and Social Care. 2021; p. 1–28. doi:10.1080/17538157.2021.1923500.
