# Supplementary material for: Understanding older adults’ perception, acceptance, and adoption of smart home technologies
Source: PLoS One. 2026 Apr 21;21(4):e0345563. doi: 10.1371/journal.pone.0345563 (PMC13098919; doi:10.1371/journal.pone.0345563)
Supplement: Attachment — Submitted filename: PLOSOne_ReviewerResponseLetter.pdf [file pone.0345563.s002.pdf]

Journal Requirements:

1. Please ensure that your manuscript meets PLOS ONE's style requirements, including those for file naming. The PLOS ONE style templates can be found at https://journals.plos.org/plosone/s/file?id=wjVg/PLOSOne_formatting_sample_main_body.pdf and https://journals.plos.org/plosone/s/file?id=ba62/PLOSOne_formatting_sample_title_authors_affiliations.pdf

> Thank you for pointing this out. We have carefully reviewed the PLOS ONE style templates and ensured that our manuscript conform to the journal's formatting requirements.

2. We note that the grant information you provided in the 'Funding Information' and 'Financial Disclosure' sections do not match.

When you resubmit, please ensure that you provide the correct grant numbers for the awards you received for your study in the 'Funding Information' section.

> Thank you for pointing this out. This project was part of Pireh Prizada's PhD research. There is no specific funder listed under the University of St Andrews or St Leonard's, so we have selected "no funder" since this project was not directly supported by a grant.

3. Thank you for stating the following financial disclosure: This was part of PHD Funded project by the University of St Andrews.

Please state what role the funders took in the study. If the funders had no role, please state: "The funders had no role in study design, data collection and analysis, decision to publish, or preparation of the manuscript." If this statement is not correct you must amend it as needed. Please include this amended Role of Funder statement in your cover letter; we will change the online submission form on your behalf.

> Thank you for this clarification. We confirm that this study was part of a PhD project funded by the University of St Andrews. The funders had no role in study design, data collection and analysis, decision to publish, or preparation of the manuscript.

4. Please remove all personal information, ensure that the data shared are in accordance with participant consent, and re-upload a fully anonymized data set. Note: spreadsheet columns with personal information must be removed and not hidden as all hidden columns will appear in the published file. Additional guidance on preparing raw data for publication can be found in our Data Policy (https://journals.plos.org/plosone/s/data-availability#loc-human-research-participant-data-and-other-sensitive-data) and in the following article: http://www.bmj.com/content/340/bmj.c181.long.

> We thank the editor for this important reminder. We have carefully reviewed the data within the manuscript and confirm that all personal information has been fully anonymised, in accordance with participant consent. We confirm that no hidden columns remain in the manuscript files.

5. If the reviewer comments include a recommendation to cite specific previously published works, please review and evaluate these publications to determine whether they are relevant and should be cited. There is no requirement to cite these works unless the editor has indicated otherwise.

> We carefully considered the reviewer's feedback. While no additional citation suggestions were provided, we have included new references to support the statements added in response to the reviewer's comments. We sincerely appreciate the reviewer's thoughtful guidance.

Reviewers' comments:

Reviewer #1:

I hope this letter finds you well. I had the opportunity to review your article titled, "Understanding older adult's perception, acceptance, and adoption of smart home technologies", which was submitted the Plos One.

1. Abstract

   – The abstract clearly suggests the need for research on the adoption of smart home technology by the elderly.
   – In particular, the fact that it clearly raised awareness of the problem in connection with changes in quality of life (QoL) after COVID-19 is a timely and academically and socially persuasive starting point.
   – It briefly explains that this is a qualitative study based on online interviews with 21 older adults, and briefly mentions the age range of the participants, region, and interview method.
   – Recommendations for smart home technology designers and researchers (e.g., affordability, environmental impact, cultural differences, etc.) are valid and have practical implications.
   – However, the results summary remains in rather general sentences and lacks quantitative or theme-focused summaries.
   – And there is a problem with the suggestion that by compressing too much content into one paragraph, the core message becomes weak.

> - **Section:** Abstract
> - **Page:** 1
>
> We sincerely thank the reviewer for the constructive feedback. We are pleased that the abstract was recognised for its clarity in emphasising the importance of studying smart home technology adoption among older adults, particularly regarding quality of life after COVID-19, and for outlining the study design and practical implications.
>
> In response to the reviewer's suggestions, we revised the abstract to:

1. Provide theme-focused highlights of the key findings rather than general statements.
2. Strengthen the results summary with clearer emphasis on major themes.
3. Restructured the abstract to enhance readability and ensure the core message stands out.

These changes are now reflected in red in the manuscript.

2. Introduction

   – The introduction presents the necessity of smart home technology as a technological solution to improve the quality of life (QoL) of the elderly, starting from the advent of an aging society and the resulting increase in the medical burden.
   – The timeliness and importance of this research are highlighted, particularly against the backdrop of social isolation and limited access to healthcare brought about by the COVID-19 pandemic.
   – There appears to be an attempt to explain the differences from previous studies by mentioning positive attitudes (e.g., references [9–14]) and factors hindering technology adoption (e.g., privacy, trust, etc.) in existing studies.
   – What is unique about the research objectives is that they describe technology adoption in a post-COVID-19 environment, cultural context (multi-country target), and interview structure centered on smart home commercial technologies.
   – The sentence structure of the introduction is clear and logically explained.
   – However, although the expressions ‘limited research’ or ‘gap’ are repeated, there is a lack of critical consideration of which aspects are not specifically addressed in which literature.
   – Although the research design includes a comparative perspective across regions and cultures (e.g., Europe vs. Pakistan), the introduction lacks a comparison with prior research on this.
   – There are many sentences that repeat the research objectives, resulting in unnecessary duplication, and it is judged that there is insufficient distinction between the purpose statement and the literature review.
   – Although it has professionalism and originality, the sentences are somewhat long and the main message is not clearly expressed.

- **Section:** Introduction
- **Page:** 2
- **Lines:** 20–47

We sincerely thank the reviewer for the detailed and insightful feedback on the Introduction. We appreciate the positive and constructive feedback regarding our manuscript.

In response to the reviewer's suggestions, we made the following revisions:

1. To address this concern, we revised the text to more clearly identify the gaps our study addresses.
2. The final paragraph of the Introduction now better reflects prior cross-cultural research and clearly state the gap our study addresses.
3. Redundancies in the statement of research objectives were removed to avoid repetition.
4. Several sentences were restructured to improve readability, enhance clarity, and highlight the main message more directly.

3. Method

– The adoption of online interviews and 'thematic analysis' as qualitative research methods clearly explains the purpose of seeking to deeply understand the elderly's perception of and acceptance of smart home technology.

– Comparative analysis that considers the cultural diversity of various countries (UK, Malta, Pakistan) is also evaluated as a strength in the design.

– The approach based on the interview language (English, Urdu, Sindhi) is considered positive as it is designed with cultural and linguistic sensitivity in mind.

– The three-stage interview structure, including online interviews via Microsoft Teams, presentation of image and video materials, Q&A, and a final questionnaire, was systematically designed.

– It is judged that the explicit description of the visual materials and interaction methods provided a real sense of immersion.

– However, the sampling method is based on volunteer responses, and there's a high possibility that the study will be biased toward older adults with digital capabilities. While the researcher acknowledged this limitation, a specific response strategy is lacking.

– In qualitative research, it is essential to mention the clarity of the analysis procedure, replicability, and reliability-enhancing strategies (RQDA, inter-rater agreement, etc.).

- **Section:** Methods, Limitations

We sincerely thank the reviewer for their careful evaluation and positive assessment of the methodological design.

In response to the reviewer's constructive suggestions, we have made the following improvements:

1. To address concerns about sampling bias, we expanded the discussion of this limitation and added a more detailed explanation. These revisions can be found in **Limitation section, Page 21, Lines 762–781**.

2. We strengthened the section by adding revised paragraph which now provides clarity on the analysis procedures and reliability considerations. This can be found on **Methodology section, Page 3, Lines 104–109**

4. Results

 – The results section is organized into 13 main topics, each with clear subheadings for easy readability.
 – Each topic includes participant quotes (direct speech), diagrams (Figures 2-8), and comparative explanations, demonstrating an effort to concretize the interpretation of qualitative data.
 – "Environmental impact," "loss of human contact," "distrust of technology," "over-monitoring," and "design issues" appear to have been extracted based on participant narratives and are compelling, realistic themes based on real-world experiences.
 – Figures 2-8 supplement the text by visually illustrating the participants' level of technical understanding, concerns (privacy, cost, etc.), and technology preferences.
 – The results section is mostly 'phenomenal descriptive' and contains many parts that are a direct summary of the participants' statements.
 – However, although the quotations are abundant, some themes are repeated without quotations, and there is a relative lack of analysis on the number and distribution of participants (country, gender, age, etc.).
 – Interpretations of cultural differences are not consistently applied across the results section, and an analytical framework that takes cultural context into account is not explicitly stated.
 – It is judged that there is a lack of theoretical explanation as to why this difference in attitude arose.

- **Section:** Results
- **Page:** 8–18

We sincerely thank the reviewer for the careful and thorough evaluation of the Results section.

In response to the reviewer's constructive concerns, we have undertaken the following revisions:

1. Across all themes, we strengthened the analysis by linking findings to relevant theoretical frameworks and references, incorporating participant numbers/distribution. We also applied consistent cross-cultural comparisons (European vs. Pakistani participants). This can be found in **Results section from pages 8 to 18**, highlighted in red throughout the section.
2. Tables 3 and 4 present the demographic distribution. Additional detail has now been provided in the **Discussion section (Page 20, Lines 721–730)**, where the demographic breakdown is discussed.

5. Discussion

- The discussion was well organized and summarized with key findings (lack of awareness, privacy concerns, affordability issues, etc.).
- The purpose and results of the study are logically explained by repeatedly emphasizing the message that 'many participants recognize the potential of the technology, but practical barriers still exist.'
- The book's strong point is that it addresses the differences in perception between Pakistani and European older adults, and it was also impressive that it included some cultural explanations.
- Provides practical advice for a wide range of stakeholders, including designers, developers, policymakers, and family members.
- It is considered positive in terms of academic integrity that the researcher himself explicitly states limitations on the number of participants (small sample), digital accessibility bias, and limitations of online interviews.
- However, although there are some connections with existing literature, it is regrettable that most of them are merely repetitions of participant statements or observational interpretations.
- Compared to the implications of the 'multicultural context' addressed in this paper, it is judged that there is a lack of systematic structuring of the influence of culture.
- The practical suggestions are abundant, but the 'theoretical contribution' is unclear.
- There is a need to improve the general tone that appears despite the sample being biased towards the UK.

- **Section:** Discussion
- **Page:** 18–20

We appreciate and thank the reviewer for the feedback on the Discussion section.

In response to the reviewer's constructive concerns, we have made the following revisions:

1. We have strengthened the discussion section by embedding our findings more explicitly within existing empirical literature.
2. We have updated the discussion to make the cultural context on smart home technology adoption, clearer between Pakistani and European participants.
3. We updated the section to connect our findings to established models of technology adoption and ageing, highlighted in red in the discussion section.
4. We have updated the section **Lines 721-724** to acknowledge our participants data more skewed towards UK's participants.

6. Conclusion

- The conclusion summarizes the key message drawn from the research results rela-

tively well—that smart home technology has the potential to improve the quality of life (QoL) of older adults, but adoption is low due to barriers such as lack of awareness, privacy concerns, and cost burden.

- Throughout the paper, this study provides empirical insights based on qualitative exploration, but the conclusion does not explain whether any new theoretical implications or conceptual extensions have been made.
- The limitations of the discussion were clearly recognized, and the conclusion also mentions the need for follow-up research on various regional, cultural, and digital competency levels.
- The sentences are professional and logical, but they are somewhat redundant and lack a definitive message.

- **Section:** Conclusion
- **Page:** 21-22

We appreciate the reviewer for their feedback on the Conclusion section.

In response to the reviewer's constructive suggestions, we have made the following improvements:

We have revised the conclusion and added modified from **Lines 793-797 and Lines 806-808** to make it more clear.

Reviewer #2:

This study explored incentives and barriers to smart home technology adoption among older adults, examining how COVID-19 affected their quality of life. Through online interviews via Qualtrics with 21 participants aged 65–90 from various countries, the study identified that limited knowledge, affordability, privacy concerns, and integration issues hindered adoption, while perceived QoL improvements motivated interest.

Major edits:

1. The five smart home technology devices discussed are primarily manufactured in the EU or USA and mainly serve the US and European markets. Participants from Pakistan, particularly rural areas, may have limited familiarity with these devices as they are not readily available in their local markets.

- **Section:** Limitaions
- **Page:** 21
- **Lines:** 772–782

We thank the reviewer for this observation and constructive feedback.

In response, we have expanded the Limitations section to note this limitation and provided a mitigation strategy for future work.

2. Based on the first point, if participants are not familiar with these devices, their feedback on the influence of QoL is also limited, affecting the interpretation of their attitudes toward smart home devices.

- **Section:** Methods, Limitations
- **Page:** 4, 21

We thank the reviewer for raising this concern.

To address this point, we have extended our **Limitation section** to address this concern on **Line 763-782, Page 21**.

In addition, we have defined the strategy adapted for participants unfamiliar with the technologies during the interview has been included in the **Methods section (Page 4, Lines 123–125).**

Minor edits:

1. Include exclusion criteria for participant selection. A flowchart showing how many participants were included/excluded based on these criteria is preferred to clarify how participants were filtered or screened for eligibility.

- **Section:** Methods, Eligibility
- **Page:** 4
- **Lines:** 131–136

We sincerely thank the reviewer for this valuable suggestion. In response, we have added a new section **Eligibility** which details inclusion and exclusion criteria. We have made it clear that all participants who signed up took part in the study.

2. Consider a paragraph or sentences of age subgroup to examine the influence of age on the perceived QoL impact of these smart home devices. For example, older adults over 80 may encounter more technical difficulties when using smart home devices in home settings.

- **Section:** Limitations
- **Page:** 21
- **Lines:** 763–768

We thank the reviewer for this suggestion. In response, we have expanded Limitaions section to include this.
