# Supplementary material for: Understanding older adults’ perception, acceptance, and adoption of smart home technologies
Source: PLoS One. 2026 Apr 21;21(4):e0345563. doi: 10.1371/journal.pone.0345563 (PMC13098919; doi:10.1371/journal.pone.0345563)
Supplement: Attachment — Submitted filename: reviewerresponse.pdf [file pone.0345563.s003.pdf]

Journal Requirements:

1. If the reviewer comments include a recommendation to cite specific previously published works, please review and evaluate these publications to determine whether they are relevant and should be cited. There is no requirement to cite these works unless the editor has indicated otherwise.

> We carefully considered the reviewer's feedback. While no additional citation suggestions were provided, we have included new references to support the statements added in response to the reviewer's comments. The following reference have been added to the paper:
>
> Ishaq E, Bashir S, Zakariya R, Sarwar A. Technology Acceptance Behavior and Feedback Loop: Exploring Reverse Causality of TAM in Post-COVID-19 Scenario. Frontiers in Psychology. 2021;12. doi:10.3389/fpsyg.2021.682507.
>
> Mitzner TL, Savla J, Boot WR, Sharit J, Charness N, Czaja SJ, et al. Technology Adoption by Older Adults: Findings From the PRISM Trial. The Gerontologist. 2018;59(1):34–44. doi:10.1093/geront/gny113.

2. Please review your reference list to ensure that it is complete and correct. If you have cited papers that have been retracted, please include the rationale for doing so in the manuscript text, or remove these references and replace them with relevant current references. Any changes to the reference list should be mentioned in the rebuttal letter that accompanies your revised manuscript. If you need to cite a retracted article, indicate the article's retracted status in the References list and also include a citation and full reference for the retraction notice.

> We thank the editor for this important reminder. All references have been checked for completeness and accuracy, and we confirm that no retracted papers have been included.

Reviewers 1 comments:

The researcher believes that many of the revisions made by the reviewers have been corrected. - However, shortcomings are still being discovered. - The specific details are as follows.

1. Further explanation of the theoretical background for TAM or UTAUT-based variables is needed.

> Thank you for pointing this out. We expanded these explanations in the section where TAM (Page 10, Lines 299-303) or UTAUT (Page 19, Lines 664-668) first appear.

2. Please clearly state the purpose of the study. (What are the determinants influencing older adults' acceptance of smart homes post-COVID.

> We thank the reviewer for this helpful comment. To clearly state the purpose of the study, we have renamed the section from "Motivation and goal" to "Mo-

tivation and Objective" to improve clarity (Page 2, Line 48). We have also expanded the section discussing the impact of COVID-19 and added relevant references (Page 9, Line 228-233). Citation Added: Ishaq E, Bashir S, Zakariya R, Sarwar A. Technology Acceptance Behavior and Feedback Loop: Exploring Reverse Causality of TAM in Post-COVID-19 Scenario. Frontiers in Psychology. 2021;12. doi:10.3389/fpsyg.2021.682507.

3. It would be a better study if we could compare the differences in perception across countries, gender, and age.

We thank the reviewer for this suggestion. A country-level comparison is presented in the Results (pp. 8–18). However, because only one participant was from Malta, we combined Malta with the UK under a broader 'Europe' category to allow a more meaningful comparison with Pakistan. We also appreciate the reviewer's interest in gender and age stratification. Consistent with standard gerontological practice, we define older adults as individuals aged 65 years and above, a threshold widely adopted in technology adoption research without further subdivision, as it reflects key life-stage transitions and ensures robust sample sizes for identifying broad patterns like digital literacy barriers. High-impact studies in this domain routinely analyse 65+ as a cohesive group to evaluate outcomes such as internet uptake and intervention efficacy (e.g., PRISM trial cited below and on page 3, Line 80-82). Therefore, stratification was not pursued here, as our objectives center on overarching trends rather than intra-group heterogeneity, and our data would yield underpowered subgroups. Gender was not a primary objective of the study, so we have acknowledged this in the limitations section (Page 21, Lines 796–799).

Reference Added: Mitzner TL, Savla J, Boot WR, Sharit J, Charness N, Czaja SJ, et al. Technology Adoption by Older Adults: Findings From the PRISM Trial. The Gerontologist. 2018;59(1):34–44. doi:10.1093/geront/gny113.

4. Please interpret the results in connection with prior research or theoretical variables that can support them in the discussion.

We thank the reviewer for the comment. We have expanded the discussion to make this clear. These revisions appear on Page 21, pages 756-764.

5. Please revise the entire thesis to have an academic structure rather than a report-style description (use thesis-style sentences and clear subparagraph topics).

Thank you for the comment. As the manuscript must strictly follow the PLOS ONE article format, we are unable to alter the structure.
