# Supplementary material for: Understanding older adults’ perception, acceptance, and adoption of smart home technologies
Source: PLoS One. 2026 Apr 21;21(4):e0345563. doi: 10.1371/journal.pone.0345563 (PMC13098919; doi:10.1371/journal.pone.0345563)
Supplement: Attachment — Submitted filename: ResponsetoReviewers_3Round.pdf [file pone.0345563.s004.pdf]

Journal Requirements:

1. If the reviewer comments include a recommendation to cite specific previously published works, please review and evaluate these publications to determine whether they are relevant and should be cited. There is no requirement to cite these works unless the editor has indicated otherwise.

> We carefully considered the reviewer's feedback. While no additional citation suggestions were provided, we have included new reference to support our statement added in response to the reviewer's comments. The following reference have been added to the paper:
>
> Galanza WS, Offerman J, Fristedt S, Iwarsson S, Malesevic N, Schmidt SM. Smart home technology to support engagement in everyday activities while ageing: A focus group study with current and future generations of older adults. PLOS ONE. 2025;20(1):1–20. doi:10.1371/journal.pone.0317352

Reviewers 1 comments:

Motivation and objectives: Why do you assume that older people has resistance towards smart homes? Recent research by Son Galanza et. al. suggests otherwise. It is important to avoid ageistic assumptions. Also the term elderly is considered stigmatising in gerontology, use older adults insteas.

> We thank the reviewer for this comment. Our findings align with the reviewer's observation, which has now been made explicit and Galanza et al. (2025) is now cited to further support this in the manuscript (Line 67-69, Page 3).
>
> The term older adults is used consistently throughout the manuscript. Elderly appears only when referring to stigmatising marketing language or when explicitly used by participants and the term is placed in quotation mark to make it clear (Line 257, page 9).

Methodology: Arguments for including people from age 65 is week and needs to be expanded. And why did you stop at age of 85?

> All participants were adults aged 65 and above, age threshold chosen to be consistent with definitions of older adults in technology-adoption research [26] (This is already present on page 3, line 81-83).
>
> No upper age limit was imposed. Participants aged 65–85 were included based on recruitment. The age range reflects the age of the oldest participant rather than an exclusion criterion, we have made this explicit in the Interviews section, page 3, Lines 89-90.

I guess that the interviews were done in respondents native language - but that needs to be written out.

> We have made this clearer in Methods section, Line 87-88, Page 3.

Why did not all participant receive an incentive (token of appreciation)?

> A small shopping voucher was provided to UK participants in accordance with local ethics approval; no incentives were offered in other contexts as it was not feasible within the approved logistical and administrative arrangements. We have made this clear on Line 134-136, page 4.

Why include only one person from Malta? How is that context comparable to UK?

> Participation was open internationally and based on voluntary recruitment; the single participant from Malta reflects uptake rather than targeted sampling. The analysis does not rely on country-level comparisons. This is mentioned already in Limitation section line 804-806.

How were the age distributions per country? Are groups similar? What about other socio-economics such as income and education that may be of relevance for findings?

> The study was not designed to match age or socio-economic characteristics across countries. Income-related differences were explored qualitatively in the affordability analysis, while country-level or stratified comparisons were not intended; this is now clarified in the Limitations section, Page 21, Lines 806-808.

Where the three phases of interviews done at same time? The interviews seemed fairly short in time. Was sufficient depth reached?

> Yes, all three phases were conducted within a single interview session. Interviews lasted approximately 45 minutes on average, which allowed sufficient depth to explore participants' experiences, perceptions, and adoption considerations across the three phases. We have made this clear on Line 156-157, Page 6.

Findings: The results includes also other references - how come? I think that the findings from this study should only be reported. As it reads now findings lack depth. The structure with other references makes it hard to identify the contribution of this study - and very few quotes are used to support the findings. Why use percentages to report findings when numbers are well below 100? The figure does not make sense as the groups seem to differ much and group belonging is not reported.

> We thank the reviewer for the comments. References are included in the Findings section to contextualise our results within the existing literature, as requested in earlier review rounds and in line with common qualitative reporting practices. All findings reported are derived from the present study; external sources are used solely for interpretive context. Both absolute numbers and percentages are provided to support transparency and aid interpretation given the sample size. Absolute numbers in addition to percentages were added following feedback from the previous round of reviews. The figure is intended to visually illustrate observed group differences, with explanations provided in the paper.

Discussion: TAM, UTAUT and DOI as theories are relevant - however rather mentioned than applied.

> TAM, UTAUT, and DOI are referenced to situate the findings within established technology adoption literature rather than to serve as analytical frameworks. These were specifically added due to helpful comments form a previous round of reviews. The study is exploratory in nature and does not aim to apply or test these models.

Overall: The populations are rather diverse while that may provide different perspectives I don't think the study reaches sufficient depth especially as the interviews had to be done online. For non-experience participants looking at pictures will not be sufficient to display adoption. And likely you are comparing two very/too different groups so I'm not sure that socio-cultural contexts and intergenerational dynamics make the difference (as written in the conclusion) but rather that the Pakistani group had very little experience, while UK clearly had more.

> We thank the reviewer for the feedback. The diversity of the sample was intentional and aligned with the study aims to capture breadth of perspectives. Interviews were in-depth (45 minutes), followed a consistent protocol, and adequately addressed the research objectives despite being conducted online. Differences in experience levels across groups and other limitations are clearly acknowledged in the manuscript. The interpretation reflects contextual and socio-cultural factors rather than direct group comparison.
